# Economic Injury Levels for Flea Beetles (*Phyllotreta* spp.; Coleoptera: Chrysomelidae) in Spring Oilseed Rape (*Brassica napus*; Brassicales: Brassicaceae)

**DOI:** 10.1093/jee/toz347

**Published:** 2019-12-27

**Authors:** Ola Lundin

**Affiliations:** Swedish University of Agricultural Sciences, Department of Ecology, Uppsala, Sweden

**Keywords:** integrated pest management, pyrethroid, canola

## Abstract

Flea beetles (*Phyllotreta* spp.) are major insect pests in spring oilseed rape (SOSR; *Brassica napus* L.). Prohibited use of three neonicotinoid insecticides in the European Union means that there are currently no insecticide seed treatment options available. Insecticide spraying remains as a control option, but there is a need to estimate the economic threshold for crop injury. As a first step to this end, economic injury levels were determined for flea beetles in SOSR. Data from 16 field experiments were used to quantify the relationship between flea beetle crop injury and SOSR yield, and additional data from paired sprayed and unsprayed plots in 12 commercial SOSR fields were used to determine the reduction in crop injury from a pyrethroid spray. There was a strong linear negative effect of flea beetle injury with 19 kg/ha yield loss per percent crop injury to seedlings and a pyrethroid spray reduced crop injury by 39%. These results gave an economic injury level of 11% defoliation of SOSR seedlings under average oilseed rape prices and insecticide use costs in 2017. This is considerably lower than previously used nominal thresholds of 25–30% injury to cotyledons. Increased yields and increasingly cheaper pyrethroids might be the reason for the lower levels of crop injury that warrant chemical control. The economic injury levels presented here can be used to construct economic thresholds that preferably should also take into account crop growth stage, crop growth rate, and anticipated flea beetle activity.

Flea beetles (*Phyllotreta* spp.) are major insect pests in spring sown oilseed rape (SOSR; *Brassica napus* L.) in both Europe and North America (Ekbom 2010, Sekulic and Rempel 2016, Knodel 2017). The most severe injury takes place when the adults that have emerged from overwintering sites move into crops to feed on seedlings during the first weeks following crop emergence (Burgess 1977, Bracken and Bucher 1986, Ulmer and Dosdall 2006). Feeding on the cotyledons and first true leaves cause characteristic injury with a shot-hole appearance, which reduce the plant’s photosynthetic capacity (Brandt and Lamb 1993, Knodel 2017). Stems, petioles, and apical meristems are also fed on, which can lead to cotyledon loss if the petiole is cut, or plant loss if the stem is cut or the apical meristem is severely injured (Brandt and Lamb 1993, Gavloski and Lamb 2000a, Knodel 2017). Flea beetle injury leads to reduced crop plant densities, delayed and uneven crop growth, seed yield loss, and increased chlorophyll content of the seed (Lamb 1984). Warm and dry weather favors flea beetle activity and crop injury while hampering crop growth (Palaniswamy et al. 1998). Flea beetle crop injury can progress quickly and lead to complete loss of the crop if not controlled (Sekulic and Rempel 2016). In Sweden, the most common flea beetle species found in SOSR are *Phyllotreta atra* Fabricius, *P. nigripes* Fabricius, *P. striolata* Fabricius, *P. undulata* Kutschera, and *P. vittula* Redtenbacher (Ekbom 1990). Insecticide seed treatments have previously been used to manage flea beetle crop injury (Ekbom and Müller 2011), but a prohibition on outdoor use of the neonicotinoid insecticides clothianidin, imidacloprid, and thiamethoxam in the European Union means that there are not currently any insecticide seed treatment options available for farmers, raising a need for research into alternative methods for pest control (EC 2018, Lundin et al. 2018).

Insecticide spraying against flea beetles remains as a viable chemical control option. Nominal thresholds for flea beetle crop injury are in place in Sweden, Finland, and Canada, which recommend that an insecticide is applied when 25–30% of the cotyledon area is injured by flea beetle feeding (Ekbom 2010). Unfortunately, there are no formal analyses of empirical data validating such recommendations. This may reflect a general paucity of published evidence for economic thresholds (Ramsden et al. 2017). In an experimental approach, a threshold of 15–20% leaf injury was suggested to balance maximized yield with a minimum of spray applications (Tangtrakulwanich et al. 2014), but the economics of such a pest management strategy were not considered. There is a need to develop economic threshold levels for Phyllotreta crop injury in SOSR.

As a first step to this end, my aim was to develop economic injury levels for flea beetles in SOSR, which take into account economic yield loss due to flea beetle crop injury, costs of insecticide sprays, and the efficacy of insecticide sprays in reducing yield loss. The economic injury level is the lowest pest density causing economic damage, whereas the economic threshold is the pest density where control measures need to be taken to prevent a pest from reaching the economic injury level (Stern et al. 1959). I used field experiments to quantify the relationship between crop injury and yield and the reduction in crop injury from a pyrethroid spray. I calculated economic injury levels using the obtained empirical results under a realistic range of oilseed prices and insecticide spraying costs.

## Materials and Methods

### Relationship between Crop Injury and Yield

To quantify the relationship between flea beetle crop injury and SOSR yield, I searched for freely accessible pesticide trial data in a Swedish online database for agronomic field trials (SUAS 2019). I found 16 experiments that reported flea beetle crop injury and SOSR crop yield (Table 1). The experiments were conducted from 2004 to 2010 in Södermanland, Stockholm, Uppsala, and Västmanland in south central Sweden. Crop injury caused by Phyllotreta flea beetles in spring oilseed rape in Sweden is greatest in this part of the country (Ekbom and Müller 2011). The trials were conducted as randomized complete block experiments with each treatment replicated in four blocks. Each experiment had three to eight treatments. In total, there were 92 experiment by treatment combinations for the 16 field experiments. Treatments consisted of various insecticide seed treatments and spray compounds that were tested for flea beetle control. In a few cases, increased seeding rates were also included as a potential alternative control measure against flea beetles (Dosdall et al. 1999, Dosdall and Stevenson 2005). Most experiments also had an untreated control. Plots were 10–12 m long and 3.5–4 m wide. Crop injury in each plot was quantified in the seedling stage when the crop had one to three true leaves by assessing the percent leaf area injured by flea beetles. The observer assessed the percent leaf area injured by flea beetles on each of 10 plants in four locations per plot. The detailed protocols for quantifying flea beetle crop injury varied slightly by experiments (Table 1); the leaf area assessed for injury was most often the cotyledons, but occasionally, it was the first pair of true leaves instead. Plots were harvested at maturity with experimental threshers, and seeds were rinsed and evaluated for water content. Yield is expressed as kg seed per hectare at nine percent water content. Both crop injury and yield data were typically available as averages per treatment in each experiment, and this is the level of aggregation that I used in statistical analyses.

**Table 1.** Details for spring oilseed rape field experiments used to determine the relationship between Phyllotreta crop injury and crop yield

Year	County	Seeded	Assessed date	Assessed BBCH	Assessed type	Harvest date
2004	Stockholm	27 April	27 May	12	True leaves	13 Sept.
2004	Uppsala	29 April	19 May	11	Cotyledons	17 Sept.
2004	Västmanland	29 April	21 May	11	Cotyledons	16 Sept.
2005	Stockholm	27 April	27 May	12	Cotyledons	5 Sept.
2005	Stockholm	n.d.	31 May	12	Cotyledons	5 Sept.
2005	Västmanland	11 May	8 June	12	True leaves	16 Sept.
2005	Västmanland	14 May	7 June	11	Cotyledons	21 Sept.
2008	Uppsala	n.d.	3 June	13	Cotyledons	n.d.
2009	Stockholm	30 April	27 May	11	Cotyledons	20 Sept.
2009	Södermanland	7 May	17 June	13	Cotyledons	26 Sept.
2009	Uppsala	22 April	27 May	12	Cotyledons	17 Sept.
2009	Västmanland	6 May	17 June	13	Cotyledons	23 Sept.
2010	Stockholm	11 May	8 June	13	Cotyledons	10 Sept.
2010	Södermanland	7 May	27 May	11	Cotyledons	3 Sept.
2010	Uppsala	11 May	8 June	12	Cotyledons	6 Sept.
2010	Västmanland	12 May	7 June	12	Cotyledons	4 Sept.

Shown for each field experiment is the year, county, date seeded, date, and crop stage when assessed for crop injury, whether cotyledons or the first pair of true leaves were assessed for injury, and the harvest date. n.d. = no data. Crop phenological development stage according to BBCH (Biologische Bundesanstalt, Bundessortenamt und Chemische Industrie; Lancashire et al. 1991).

To examine how crop injury affected yield I analyzed data in a general linear mixed model (Proc Mixed) in SAS 9.4 for Windows (SAS Institute Inc., Cary, NC). Initial inspections of the data indicated that the slope of the relationship between crop injury and yield was negative in 15 of the 16 experiments. However, the magnitude of the slope coefficient varied considerably between experiments. I accounted for this by allowing for a random slope coefficient for each experiment (see below). Yield was the response variable and the percent crop injury was the explanatory variable in the model. Year and experiment identity were added as random intercepts and percent crop injury within experiment as a random slope. I assumed normal error distribution and assessed the assumption by inspecting residual plots. Degrees of freedom were estimated with the Kenward–Roger method. I used the regression coefficient of the model as a measure of yield loss per percent increase in flea beetle crop injury. I also tested two alternative models that allowed a nonlinear yield response to crop injury (Dyer et al. 1993). First, I added a second-order (quadratic) term for crop injury. Second, to explore whether the crop could compensate for low to moderate levels of crop injury, I also tested a first-order linear term for crop injury but including only plots where the crop injury was 30% or lower.

### Insecticide Spray Efficacy

I determined the proportional reduction in flea beetle crop injury resulting from insecticide treatment with a pyrethroid spray in paired unsprayed and sprayed plots in 12 commercial SOSR fields 2017–2018 (Table 2). The 16 field experiments that were used to quantify the relationship between crop injury and crop yield were not useful for this part of the study for two reasons. First, repeated pyrethroid applications against flea beetles were typically performed in treatments assigned to receive treatment with pyrethroids, which meant that it was not possible to evaluate to what extent a single pyrethroid application reduced crop injury. Second, the restricted plot sizes in those experiments (35–48 m^2^) meant that flea beetles could easily disperse between untreated and treated plots, which would lead to an underestimation of the spray efficacy in a commercial field. In each of the 12 fields used to evaluate pyrethroid spray efficacy, two 50-m-long and approximately 24-m-wide study plots (exact width of plot corresponded to the sprayer boom width used by the farmer) were established adjacent to each other in a commercial SOSR field. One plot was treated with a pyrethroid insecticide once against flea beetles 2–5 wk after seeding as per standard field management by the farmer, whereas the other study plot was left untreated (Table 2). Crop injury caused by flea beetles was assessed in each study plot when the crop had approximately two fully developed true leaves. Injury to cotyledons was visually observed and assessed on five plants in a row at 10 locations along a 40-m transect centered in each plot. Flea beetle injury was classified into five categories: 0 = 0% of cotyledon area injured, 1 = 1–10%, 2 = 11–30%, 3 = 31–60%, and 4 = 61% or more of cotyledon area injured (Ekbom and Kuusk 2005). The classifications of cotyledon injury were converted to percentages using the center point in each injury class (0 = 0, 1 = 5.5%, 2 = 20.5%, 3 = 45.5% and 4 = 80.5%) and averaged per transect prior to statistical analyses. To examine how the pyrethroid spray affected crop injury I analyzed data in a general linear mixed model (Proc Mixed) in SAS 9.4. Percent crop injury was the response variable and insecticide spray (yes or no) and year (2017 or 2018) were categorical explanatory variables. Field was added as random factor to the model. I assumed normal error distribution and assessed the assumption by inspecting residual plots. Degrees of freedom were estimated with the Kenward–Roger method.

**Table 2.** Details for spring oilseed rape fields used to determine efficacy of insecticide treatment with a pyrethroid against Phyllotreta flea beetles

Year	County	Seeded	Sprayed	Compound	Dose	Assessed
2017	Stockholm	19 April	17 May	λ-Cyhalothrin	10	31 May
2017	Stockholm	21 April	23 May	λ-Cyhalothrin	6.25	2 June
2017	Uppsala	20 April	4 May	τ-Fluvalinate	48	3 June
2017	Uppsala	5 May	19 May	β-Cyfluthrin	7.5	9 June
2017	Uppsala	26 May	17 June	τ-Fluvalinate	48	25 June
2018	Stockholm	14 May	28 May	τ-Fluvalinate	48	5 June
2018	Stockholm	24 May	14 June	λ-Cyhalothrin	7.5	21 June
2018	Stockholm	22 May	14 June	τ-Fluvalinate	60	21 June
2018	Uppsala	30 April	20 May	β-Cyfluthrin	7.5	30 May
2018	Uppsala	16 May	19 June	τ-Fluvalinate	48	28 June
2018	Uppsala	14 May	30 May	τ-Fluvalinate	48	14 June
2018	Västmanland	7 May	23 May	λ-Cyhalothrin	7.5	1 June

Shown for each field is the year, county, date seeded, date, compound (active ingredient), and dose sprayed (g/ha), as well as the date when crop injury was assessed.

### Economic Injury Levels

I assumed that there was one economic benefit, avoided yield loss, and two economic costs, one for purchasing the insecticide and one for applying it, associated with insecticide spraying when calculating economic injury levels for flea beetles. I assumed that there were no driving damage costs to the crop from applying the insecticide. This is reasonable as crops are generally less sensitive to driving damage from applying pesticides early in the season, i.e., when treatments against flea beetles are done, compared with closer to crop maturity (Nilsson et al. 1981). Due to lack of data, I also disregarded potential effects of spraying on crop quality. The economic injury level (EIL) was calculated as follows:

$$\begin{array}{l} EIL=\frac{C}{V\cdot D\cdot K} \end{array}$$ (1)

where *C* is the cost for purchasing and applying an insecticide per hectare, *V* is the price of crop per kg, *D* is the slope of the relationship between crop injury and crop yield (crop damage per unit injury), and *K* is the proportional reduction in crop injury achieved by applying the insecticide (Pedigo et al. 1986). I used values of *D* and *K* from the empirical data collected (see sections Relationship between Crop Injury and Yield, and Insecticide Spray Efficacy). Because this EIL is expressed as a level of injury, and not as an insect population density, I exclude in equation 1 one variable in the general EIL equation, *I*, which is specifying the relationship between pest density and crop injury (Pedigo et al. 1986, Hutchins et al. 1988). I used publicly available Swedish economic data for crop and insecticide prices converted to Euros, applying an exchange rate of 10 SEK per Euro (Table 3). I calculated the ‘current’ economic injury level using the latest available oilseed rape price and insecticide costs from 2017 (Table 3). I also calculated economic injury levels for two alternative scenarios to explore how sensitive the economic injury levels were to changes in prices and costs (Table 3). In the low and high economic injury level scenario, I used the highest and lowest oilseed rape price in the last 5 yr, respectively. I also arbitrarily reduced and increased insecticide purchasing and application costs by 25% in the low and high economic injury level scenarios, respectively.

**Table 3.** Economic data for insecticide purchase cost, *C*(*insecticide*), insecticide application cost, *C*(*application*), and crop price, *V*, used for economic injury level (EIL) calculations

	Scenario		
Variable	Current	Low EIL	High EIL
*C*(*insecticide*)	5.0 €/ha	3.8 €/ha	6.3 €/ha
*C*(*application*)	20 €/ha	15 €/ha	25 €/ha
*V*	0.314 €/kg	0.333 €/kg	0.271 €/kg
EIL	11%	8%	16%

Publicly available Swedish economic data for crop and insecticide purchasing prices compiled by the Swedish Rural Economy and Agricultural Societies were used (SREAS 2019). The current average insecticide application cost was obtained from the Swedish Board of Agriculture (SBA 2018). The calculated economic injury level (EIL; percent seedling defoliation by flea beetles) for each scenario is presented in the bottom row.

## Results

### Relationship between Crop Injury and Yield

**Fig. 1.**
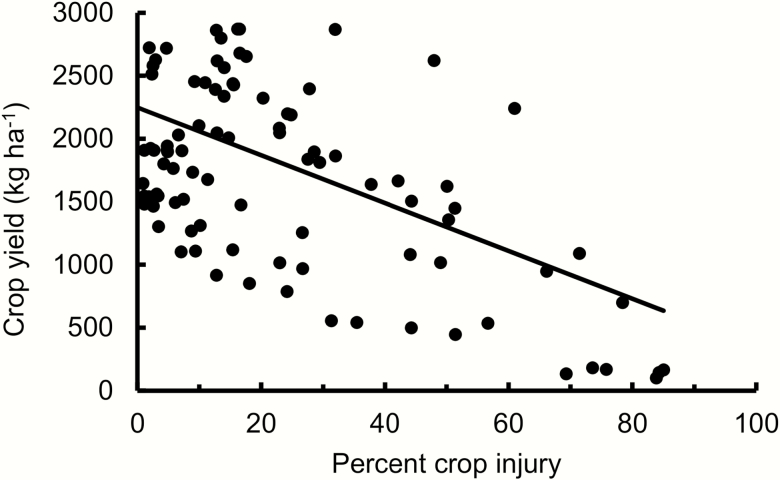
Spring oilseed rape yield in relation to percent crop injury to seedlings caused by Phyllotreta flea beetles in 16 field experiments 2004–2010. The solid line indicates the linear model prediction which was used for calculation of economic injury levels (*F*_1, 11.6_ = 26.98, *P* < 0.0010, *y* = 2,249 − 18.99*x*).

Percent crop injury by Phyllotreta flea beetles had a strongly linear negative effect on crop yield (*F*_1, 11.6_ = 26.98, *P* < 0.0010, Fig. 1). The slope (*D* in equation 1) was −18.99 kg/ha per percent crop injury. The quadratic term was not statistically significant (*F*_1, 67.8_ = 0.34, *P* = 0.56), and percent crop injury still had a significantly negative linear effect of a similar magnitude on crop yield when only including levels for crop injury up to 30% (*F*_1, 20.7_ = 10.24, *P* = 0.0044, *y* = 2,437 − 21.40*x*).

### Insecticide Spray Efficacy

A pyrethroid spray reduced the average percent crop injury from 37.2% (SE 6.5%) in unsprayed plots to 22.8% (SE 6.5%) in sprayed plots (*F*_1, 11_ = 11.64, *P* = 0.0058). Consequently, the proportional reduction in crop injury (*K* in equation 1) was estimated to 0.39.

### Economic Injury Levels

Applying *D* = 18.99 and *K* = 0.39 together with the economic input data in Table 3 to equation 1, using crop price for 2017 and assuming average insecticide treatment costs, resulted in an economic injury level of 11% crop injury to SOSR seedlings (Table 3). The calculated economic injury levels varied from 8% under the assumption of high crop price and low insecticide treatment costs, to 16% under the assumption of low crop price and high insecticide treatment costs (Table 3).

## Discussion

I quantified a strong linear negative relationship between crop injury to SOSR seedlings caused by Phyllotreta flea beetles and crop yield. I found little support for nonlinear yield response to crop injury, particularly compensation for low levels of injury. This was somewhat unexpected as oilseed rape is known to be able to compensate for considerable amounts of insect pest injury (Williams and Free 1979). Such compensation, however, is mainly documented in relation to later season injury to flowers and buds, and the degree to which plants can compensate for injury to seedlings caused by Phyllotreta feeding might be more limited (Gavloski and Lamb 2000a,b). Negative linear relationships between Phyllotreta crop injury and crop yield with a similar magnitude have also been identified in previous studies (Soroka et al. 2008, Tangtrakulwanich et al. 2014). A potential limitation when it comes to the applicability and generality of this result is that the experiments I used to determine the relationship between crop injury and yield were performed with inbred cultivars. Although inbred cultivars still are grown in Sweden to some extent, hybrid cultivars have largely replaced inbred cultivars on the market in the last few years. However, despite early hopes that hybrid cultivars were going to be less affected by flea beetle injury, the response of inbred and hybrid cultivars to crop injury by Phyllotreta are similar (Bodnaryk et al. 1994). This indicates that my results are applicable also to modern hybrid cultivars.

Pyrethroid sprays were able to reduce percent crop injury from on average 37.2 in unsprayed plots to 22.8 in sprayed plots, a reduction of 39%. A single pyrethroid spray might therefore not be sufficient for controlling heavy flea beetle attacks. While applying insecticide at the time of seeding, e.g., as seed treatments, possibly offers more effective control of flea beetle crop injury (Weiss et al. 1991), such a tactic makes it difficult to apply insecticides based on economic threshold levels. For this, it would be necessary to develop a forecasting tool that is able to predict, already when seeds are ordered, if seed treatments will be economically motivated and only use them in such cases (Douglas and Tooker 2015). Such forecasts are, however, currently not available for Phyllotreta flea beetles (Sekulic and Rempel 2016). Combining monitoring with use of spray insecticides when economic thresholds are exceeded is therefore a viable, and in Sweden and other places lacking registered insecticide seed treatments, necessary alternative for flea beetle control in SOSR. Insecticide spraying based on economic thresholds also opens up the possibility of using alternative spray biopesticides, such as entomopathogenic nematodes or fungi for Phyllotreta pest control (Reddy et al. 2014, Antwi and Reddy 2016, Briar et al. 2018).

The strong negative effect of flea beetle crop injury on crop yield, coupled with an inexpensive control measure in the form of pyrethroid spraying, resulted in an economic injury level of just 11% crop injury to SOSR seedlings based on 2017 crop price and assuming average insecticide treatment costs. The economic injury level ranged from 8 to 16% depending on crop price and insecticide treatment costs. Although further work is needed to develop economic thresholds based on the economic injury levels presented here, I suggest to apply these economic injury levels as a starting point for economic thresholds. This is reasonable considering that the economic injury level sets the maximum possible value of the economic threshold. The economic injury levels presented here are considerably lower than previously published nominal thresholds at 25–30% flea beetle injury to cotyledons (Ekbom 2010) for a wide range of crop prices and insecticide treatment costs. Since I am unaware of any calculations underlying these older thresholds, it is not possible to identify the reasons why I obtained generally lower levels of crop injury causing economic damage, but increased yields and access to increasingly cheaper pyrethroids in the last decades (Dewar 2016) might have shifted the economic injury levels downwards over time.

Applying lower thresholds might lead to increased use of spray insecticides in SOSR. As all currently registered insecticides against flea beetles in Sweden are pyrethroids, the potential risk for development of insecticide resistance in flea beetles (Turnock and Turnbull 1994, Ekbom and Müller 2011) should be considered and monitored. There is also scope to develop economic thresholds levels for Phyllotreta crop injury that take into account crop growth stage, crop growth rate, and anticipated flea beetle activity. The exact timing and speed of flea beetle defoliation relative to crop growth will probably affect threshold levels. For example, the economic threshold is likely to be considerably higher for attacks by flea beetles when the crop has started to develop the first pair of true leaves compared with an economic threshold for early attacks when the crop is still emerging. Similarly, it might not be necessary to apply an insecticide if crop injury is at thresholds levels, but crop growth is rapid and further flea beetle activity limited. As flea beetle crop injury is weather dependent and promoted by warm and dry weather (Burgess 1977, Knodel 2017), it can be tested to what extent weather forecasts can predict further development of crop injury. Parallel to future development of threshold-based control of flea beetles with insecticide sprays, further studies are also warranted that explore effective and practically applicable cultural control methods, such as altered time of seeding or tillage regimes (Knodel 2017, Lundin et al. 2018, Lundin 2019), which can reduce the need for insecticide use in SOSR and forward integrated pest management.
